# Neutron instrument concepts for a high intensity moderator at the European spallation source

**DOI:** 10.1038/s41598-024-59506-5

**Published:** 2024-04-23

**Authors:** Stavros Samothrakitis, Mads Bertelsen, Peter K. Willendrup, Erik B. Knudsen, Camilla B. Larsen, Nicola Rizzi, Luca Zanini, Valentina Santoro, Markus Strobl

**Affiliations:** 1 Applied Materials Group, Paul Scherrer Institute, Villigen, Switzerland; 2 European Spallation Source ERIC, Lund, Sweden; 3 Department of Physics, Technical University of Denmark, Copenhagen, Denmark; 4 Copenhagen Atomics, Tårnby, Denmark; 5 Niels Bohr Institute, University of Copenhagen, Copenhagen, Denmark

**Keywords:** Condensed-matter physics, Techniques and instrumentation, Condensed-matter physics, Techniques and instrumentation

## Abstract

In the course of the Horizon 2020 project HighNESS, a second moderator concept has been developed for the European Spallation Source, which complements the currently built moderator and is optimized for high intensity with a large viewable surface area. In this work we introduce conceptual designs for neutron instruments for condensed matter research designed to make optimal use of the capabilities of this moderator. The focus is on two concepts for small-angle neutron scattering and one neutron imaging instrument, which are intended to complement corresponding instruments that are already under construction at the European Spallation Source. One small-angle neutron scattering instrument concept resembles a conventional pinhole collimator geometry and aims to profit from the proposed second moderator by enabling to illuminate larger samples and providing particularly high resolution, drawing on a 30 m collimation and corresponding detector distance. A second small-angle neutron scattering instrument concept adopts nested mirror optics that enable to efficiently exploit the large moderator size and provide high resolution by focusing on the detector. The neutron imaging instrument concept is a typical pinhole instrument that can be found at continuous sources and draws on the corresponding strengths of high flux and large homogeneous fields-of-view, while still providing moderate wavelength resolution for advanced imaging methods.

## Introduction

**Figure 1:**
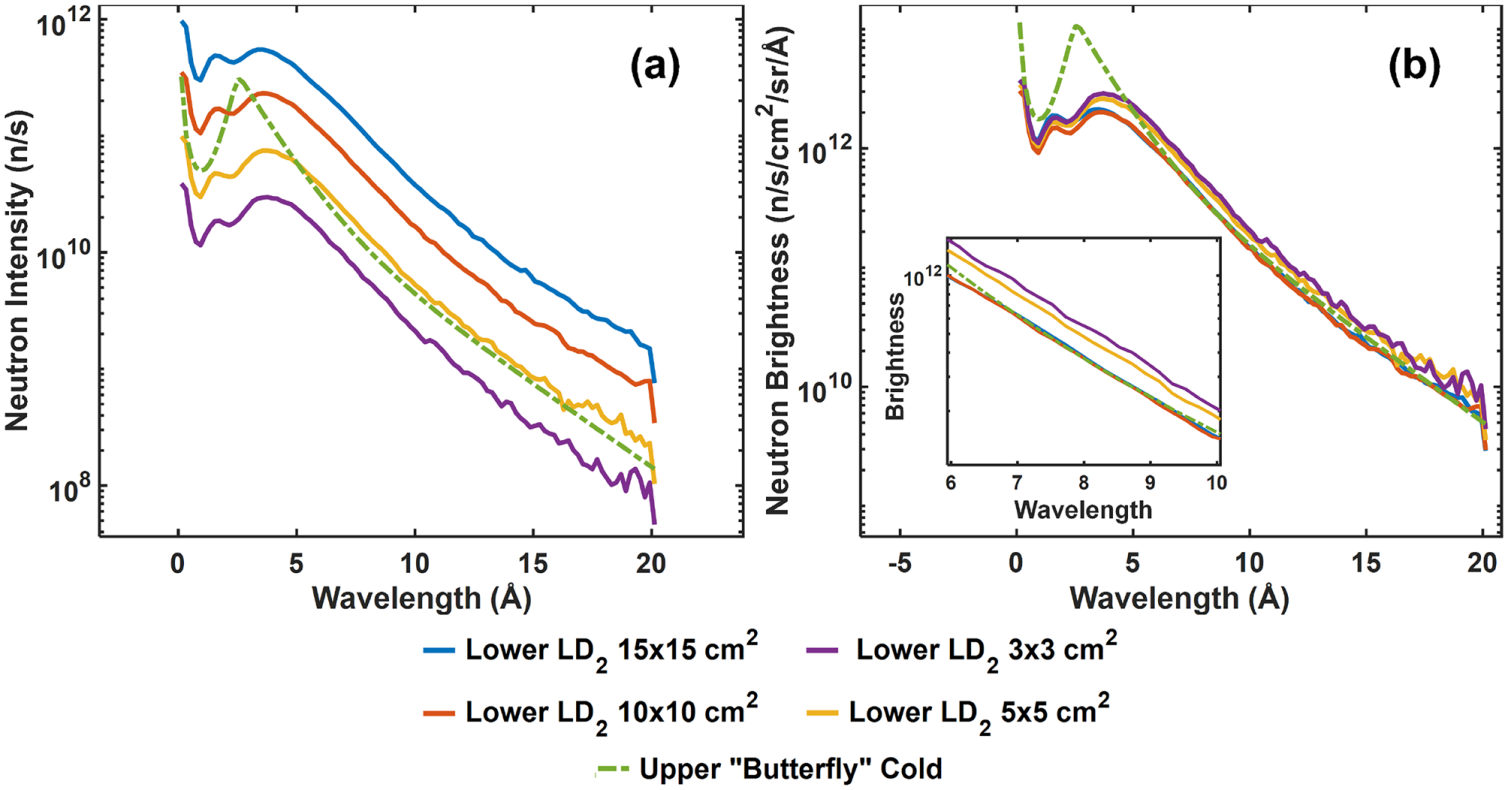
Wavelength spectra of the proposed lower liquid deuterium moderators, in terms of (**a**) neutron intensity and (**b**) neutron brightness, measured at 2 m distance, over the beam-port of the ESS monolith wall. The corresponding cold spectrum of the upper “butterfly” moderator is superimposed for comparison.

The European Spallation Source (ESS), currently under construction in Lund, Sweden, will make a suite of 15 state-of-the-art neutron scattering instruments^1^ available to the international condensed matter and neutron science communities. These instruments will be served by a moderator placed above the spallation target^2,3^. This upper moderator has a flat geometry limiting the height of the viewable moderator surface to 3 cm, enabling it to provide outstanding brightness^2,3^. However, the current infrastructure of ESS allows a second moderator to be constructed and positioned below the spallation target. This second moderator was considered in the EU Horizon 2020 project HighNESS to have a larger viewable area and to offer higher intensities with particular intensity gains at long wavelengths^4–6^. Also, very cold (10–40 Å), and ultra cold (> 100 Å) neutron sources are under consideration^6–8^. Within the HighNESS project, the moderator optimization process arrived at a large liquid deuterium vessel and different sizes of the openings for neutron extraction, to be viewed by condensed matter instruments, have been considered. Designs for four different opening sizes of 3 $\times$ 3 cm^2^, 5 $\times$ 5 cm^2^, 10 $\times$ 10 cm^2^, and 15 $\times$ 15 cm^2^ have been investigated. In Fig. 1 the wavelength spectra, in terms of neutron intensity and brightness, of these four moderators are plotted along with the corresponding spectrum for the cold part of the upper “butterfly” moderator. While the smaller openings exhibit higher brightness, this effect is much smaller for the deuterium moderator than for the hydrogen-based “butterfly”, meaning that differences in brightness are less pronounced^6^. It is also seen that for wavelengths larger than 5 Å the brightness of the larger openings is comparable to that of the “butterfly” moderator, while the two smaller openings result in only slightly higher brightness (see inset of Fig. 1b). However, the larger extraction windows (> 5 $\times$ 5 cm^2^) offer a significantly higher neutron intensity when compared to the “butterfly” moderator. As such, for the scope of the current study, the moderator with the largest extraction window, with a viewable area of 15 $\times$ 15 cm^2^, is considered as the optimal choice and basis for the presented instrumentation considerations.

Several instruments for condensed matter research can be considered to benefit from the large-area, high-intensity moderator offering a cold spectrum. Small-angle neutron scattering (SANS), neutron imaging (NI), neutron spin-echo, and time-of-flight spectrometers have been identified as prime candidates able to profit from the larger moderator surface and higher intensity in the cold wavelength regime. More specifically, SANS can profit from a larger high-intensity moderator providing a cold spectrum when considering larger samples, requiring a longer instrument, which in turn can also be used for higher resolution measurements. Alternatively, novel nested mirror optics (NMO) concepts such as Wolter optics^9–12^have been considered in order to harvest neutrons from the large moderator face and focus them on the detector in a relatively short instrument. Spin-echo is considered to profit from a similar strategy when focusing on the sample position, while e.g. a cold time-of-flight spectrometer might benefit from the large moderator face by providing a larger vertical divergence and correspondingly higher intensity to the instrument. For NI the larger moderator surface enables a large and homogeneous field-of-view (FoV) in a common pinhole geometry and a conventional short instrument featuring high flux conditions.

In this work we present three initial conceptual designs for instruments viewing this second high-intensity moderator, namely two SANS instrument concepts and one NI instrument, designed to complement instruments that are currently under-construction, such as LoKI, SKADI, and ODIN^1,13–16^.

## Small-angle neutron scattering

The conceptual designs presented for SANS aim to complement the two instruments under-construction at ESS, LoKI and SKADI, in reaching lower minimum scattering vector values $Q_{min}$ while maintaining high resolution, providing access to larger length-scale structures and thus studies of, for example, mesoscopic aggregates^17^, biological macromolecules such as RNA-protein complexes^18^, type-II superconductors featuring flux line lattices^19^, as well as skyrmion lattices and noncollinear spin structures^20,21^. A large total *Q*-range coverage is beneficial to studying multi-scale structures, including complex fluids^22^, nanocomposites^23^, and hierarchical materials^24^ containing features spanning a broad range of length scales. The possibility to maintain high flux at the sample position, in particular for a focusing SANS concept, will support dynamical studies of structural changes in materials over time, such as phase transitions^25^, kinetics of chemical reactions^26^, protein folding/unfolding dynamics^27^, and reorientation dynamics of anisometric magnetic particles^28^. Furthermore, combining the large *Q*-range and high resolution, self-assembly and growth of structures can be investigated towards longer length-scales, where many conventional SANS instruments reach their limits. Additionally, the proposed instruments can exploit extra gains in efficiency when large samples can be made available, which is the case in many fields from soft matter research, engineering materials to geology and energy research, to name but a few.

### Conceptual designs

#### ConvSANS

**Figure 2:**
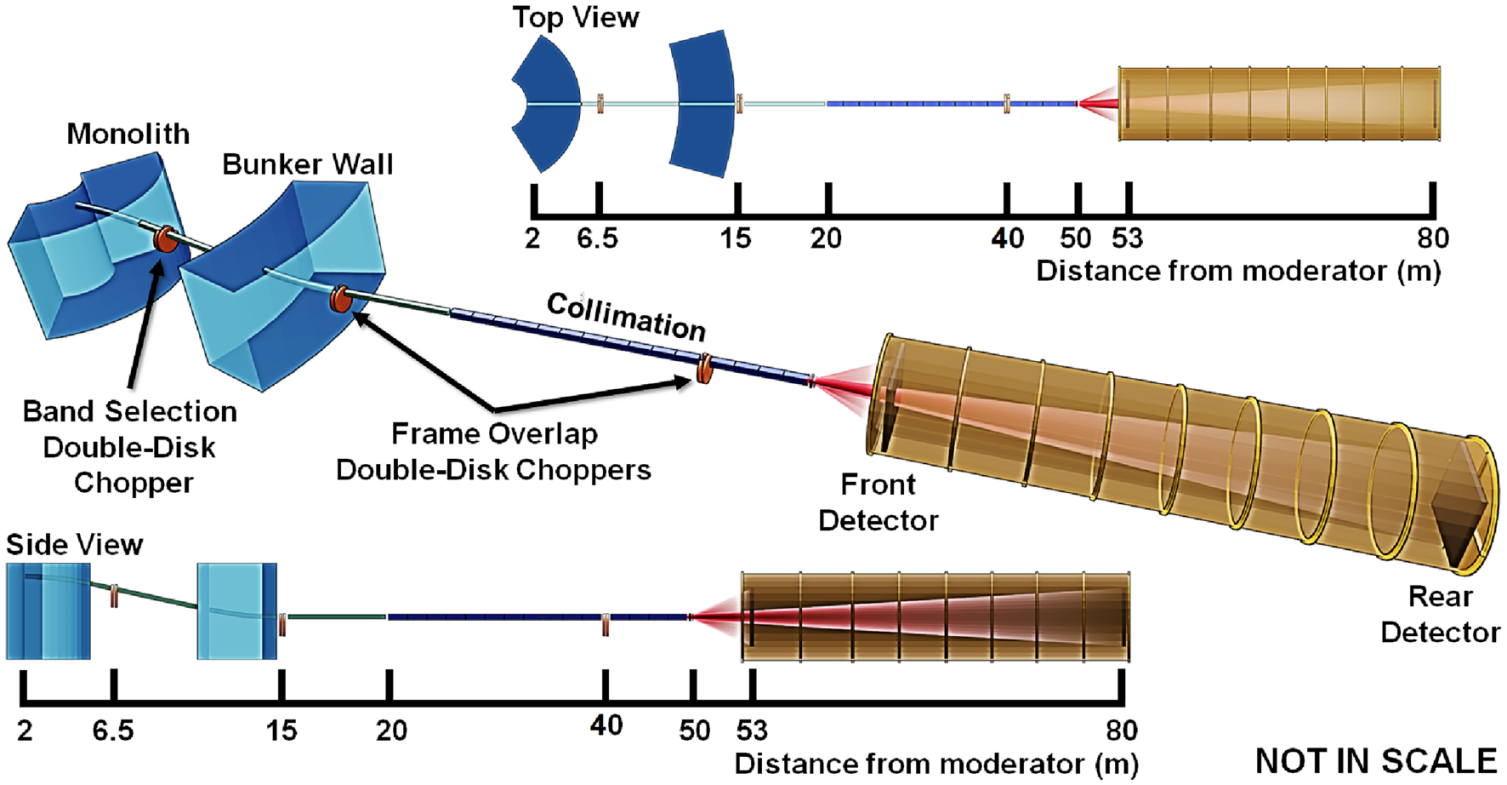
Schematic overview of the ConvSANS design in isoprojection, as well as from top and side view.

The conceptual design of a high-resolution conventional pinhole collimation SANS instrument (ConvSANS) is schematically outlined in Fig. 2.

The present instrument design assumes a constant guide size of 6 $\times$ 6 cm^2^ throughout the instrument, with the start of the neutron guide at 2 m from the moderator surface, at the inner ESS monolith wall. The instrument makes use of a pair of benders located in the monolith and the bunker wall, respectively, that are used to avoid direct line-of-sight to the moderator and act as a short wavelength cut-off filter. Similar to the SANS instrument LoKI^1^, currently under construction at the ESS, the benders are designed with a radius of curvature of 61.25 m and a length of 3.5 m. The use of the bender pair provides a twice out of line-of-sight condition to minimize the intrinsic background, and offsets the beamline down vertically by 0.545 m. Within the ESS shielding bunker two tilted straight neutron guides, 1 m and 5 m long, respectively, connect the two benders, with a gap between them reserved for chopper installation. After the second bender, a straight guide of 5 m in length and back in horizontal direction connects the bender with the collimation/slit system. From the outcome of initial Monte Carlo simulations, all neutron guides and benders are coated with *m* = 4 supermirrors, however, further studies will be performed to determine the optimal *m* value.

**Figure 3:**
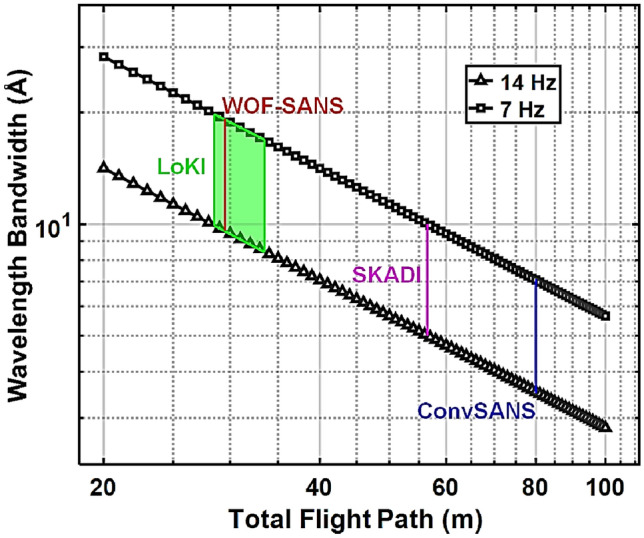
Wavelength bandwidth of ConvSANS and WOF-SANS instruments as a function of the total flight path for normal mode of operation (14 Hz) and pulse-skipping mode of operation (7 Hz). The bandwidth of the SANS instruments LoKI and SKADI are also given for comparison. Since for ConvSANS, WOF-SANS, and SKADI the longest sample-to-detector distance is fixed, the wavelength bandwidth is also fixed.

The current design features a 30 m long collimation section (*L*1). In a practical realization the collimation will consist of sections to enable to relax collimation/resolution and adapt to different sample sizes. The 30 m collimator starts at 20 m from the moderator, where a first aperture, hereafter referred to as source aperture, is located and ends at 50 m from the moderator, where the sample aperture is located. The subsequent sample area provides sufficient space for installing standard sample environment equipment. For initial characterization and assessment of performance, two fixed-position detectors are placed at 3 m and 30 m from the sample, respectively. The combination of the long collimation with the two detector positions, will allow for a large *Q*-range coverage, allowing at the same time to maintain high *Q* resolution for relatively large sample sizes. The long total instrument length of 80 m results in relatively high wavelength resolution, while the long collimation provides high angular resolution. The total length of the instrument limits the wavelength bandwidth to about 3.5 Å  when operating in the standard 14 Hz mode, however this can be extended to about 7.0 Å in the pulse skipping 7 Hz mode of operation (see Fig. 3). In addition, if required, a mode of continuously scanning over a wide wavelength bandwidth can be considered. Thus, the instrument in principle provides significant flexibility also for single shot large *Q*-range measurements at high resolution.

**Figure 4:**
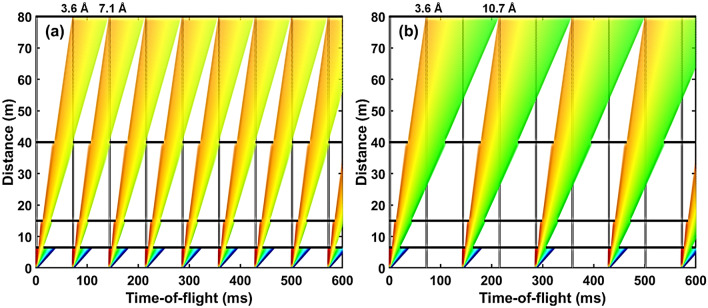
Examples of time-distance diagrams for ConvSANS showing available wavelength selection of (**a**) 3.6 Å  – 7.1 Å  for the standard 14 Hz mode of operation and (**b**) 3.6 Å  – 10.7 Å  for the pulse-skipping 7 Hz mode of operation. The vertical gray lines indicate the pulses whereas the horizontal black lines indicate the choppers at their respective positions.

Wavelength selection and frame overlap prevention is performed using two double-disc choppers. A double-disk bandwidth chopper is placed in the ESS in-bunker section of the instrument, between the two tilted neutron guides, at 6.5 m from the moderator. An additional pair of choppers is placed right after the ESS bunker wall, at 15 m, downstream of the second bender, to suppress frame overlap. A third pair of choppers can also be added further downstream, at 40 m within the collimation section, to further suppress frame overlap. The chopper function is illustrated in the time-distance diagrams of Fig. 4.

The detector configuration employed for the current setup is based on a “window-frame” design (see Fig. 2). The front detector is at a fixed position, 53 m from the moderator and 3 m from the sample position. It has a 3.0 $\times$ 3.0 m^2^ surface area with a 0.354 $\times$ 0.354 m^2^ window opening at the center. The rear detector, also at fixed position, is located at 80 m from the moderator, i.e. 30 m from the sample position, with a 3.0 $\times$ 3.0 m^2^ surface area as well, offering significant *Q*-range coverage with high resolution. In the current design, there is no specific detector technology considered (e.g. ^3^He gas detector or ^10^B-based detector).

#### WOF-SANS

**Figure 5:**
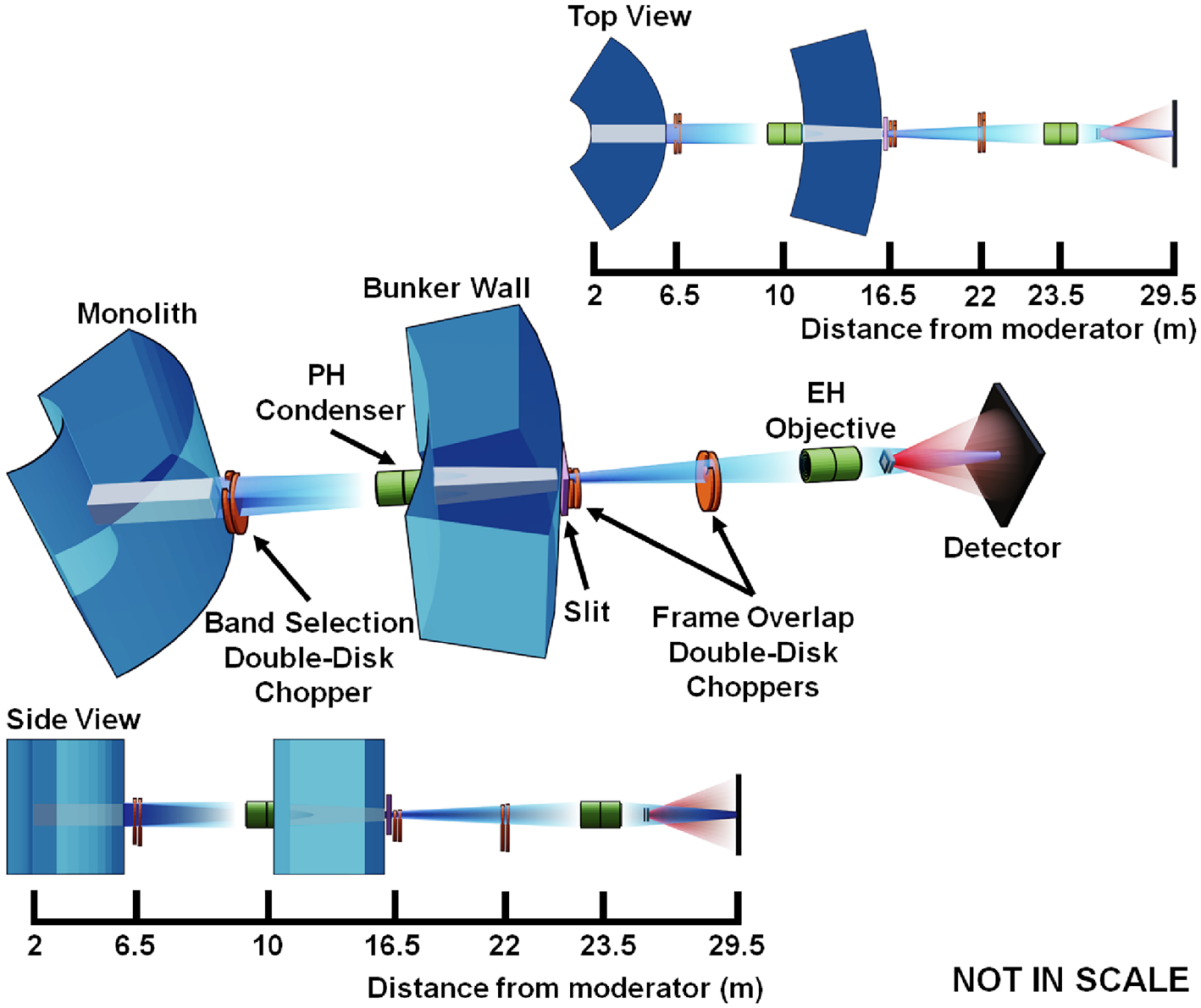
Schematic overview of the WOF-SANS design in isoprojection, as well as from top and side view.

A Wolter Optics Focusing SANS (WOF-SANS) design, as an exemplary study of using NMO for SANS, makes use of a pair of Type I Wolter optics^29^ to take advantage of the large moderator surface and thus maintain a high total neutron intensity at the sample position. A schematic of the considered instrument design can be seen in Fig. 5. The total length of the instrument is 29.5 m defining a wavelength bandwidth of 9.5 Å when operating with the 14 Hz source frequency. Again, this range can be extended to about 19 Å in a pulse skipping mode (see Fig. 3).

The first Wolter optics system is used as a condenser lens and it is located in the ESS in-bunker section of the instrument, 10 m from the moderator. In order to optimize transmission and focusing, the initial design consists of 28 cylindrical nested paired parabolic and hyperbolic (P-H) sections, currently 1.3 m and 1.0 m in length, respectively, with a total radius of 7.5 cm and a total length of 2.3 m. The innermost radius of 1.0 cm is not coated and might be filled with absorber material to avoid direct line of sight of an unfocused central beam. The focal length of the condenser is 5.5 m thus its focal point is 15.5 m from the moderator. The use of parabolic mirrors on the incoming beam side implies that predominantly the lowest divergence component of the phase space will be focused. The mirrors are currently considered coated with an *m* = 3.0 supermirror coating, i.e. with 3 times larger total reflection angle than natural Ni. An aperture of 4 mm in diameter is placed outside the bunker wall, at the focal point of the condenser, to suppress any out-of-focus rays and any neutrons that pass through the condenser without reflecting. This position will be heavily shielded and the aperture will be designed to suppress the large diameter beam of background radiation from a relatively large extraction channel from the source. A correspondingly shaped bunker wall transition channel will support this background suppression.

The second Wolter optics system is used as an objective lens and is located 23.5 m from the moderator. Its focal lengths, aperture-to-objective (*Fo*) and objective-to-detector (*Fd*), are 8 m and 6 m, respectively, resulting in a (de)magnification *M* = *Fd*/*Fo* = 0.75. As such, the beam spot size on the detector center is expected to be 3 mm in diameter. The objective lens consists of 25 nested paired elliptical and hyperbolic (E-H) sections, 0.9 m and 0.82 m in length, respectively, adding up to a total length of the lens of 1.72 m. Its maximum radius is 10 cm, allowing it to collect the full divergence coming from the aperture, i.e. from the condenser. The E-H mirrors are also coated with supermirror of *m* = 3.0, however, again this is subjected to optimization. The sample is placed between the objective lens and the detector. Different positions are possible with corresponding impact on illuminated sample size (beam size), *Q*-range, and resolution. To maintain the highest possible resolution and lowest possible $Q_{min}$ the sample can be positioned right after the objective lens. As the beam is very large in this position, most samples will use only a limited fraction of the beam in an off-axis position. This implies that for a final design other NMO designs^30,31^ apart from the the concentric Wolter optics should be considered carefully.

**Figure 6:**
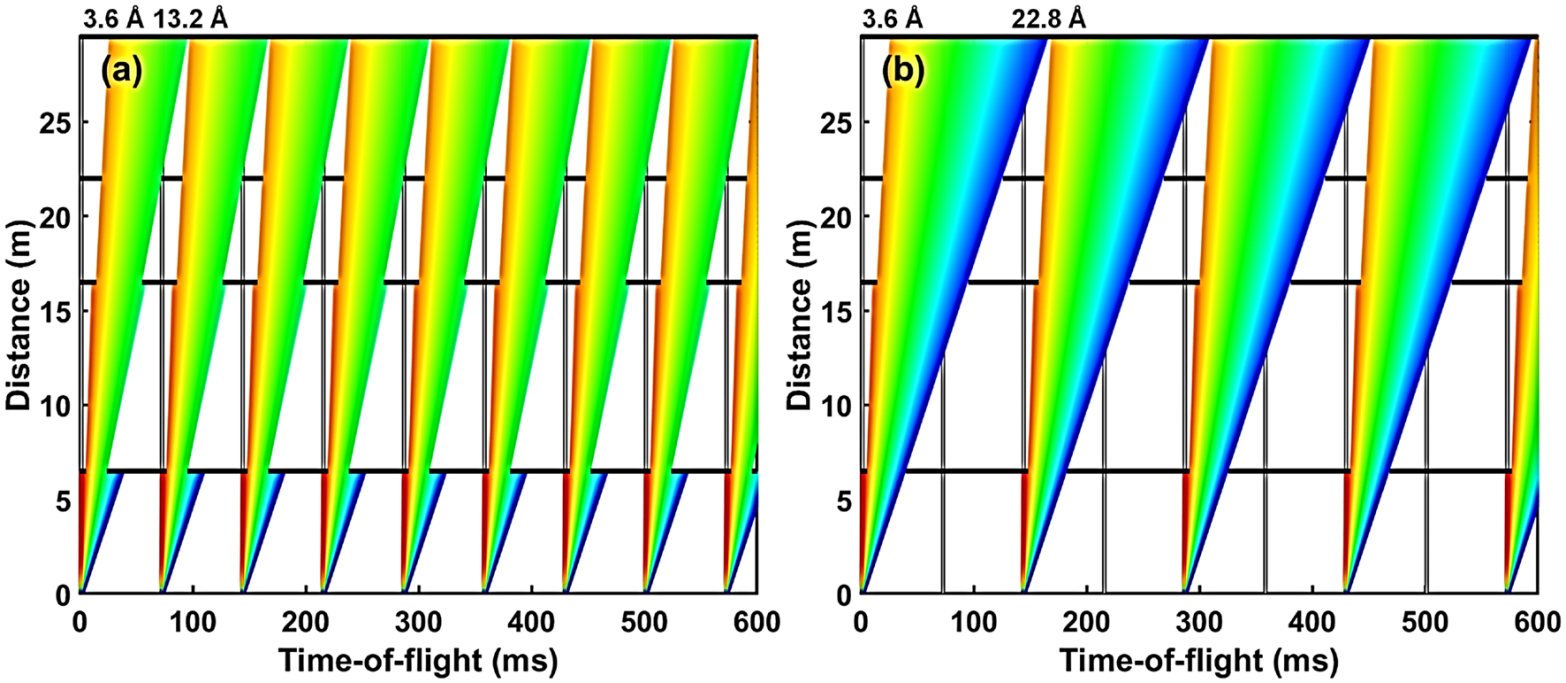
Examples of time-distance diagrams for WOF-SANS showing available wavelength selection of (**a**) 3.6 Å  – 13.2 Å  for the standard 14 Hz mode of operation and (**b**) 3.6 Å  – 22.8 Å  for the pulse-skipping 7 Hz mode of operation. The vertical gray lines indicate the pulses whereas the horizontal black lines indicate the choppers at their respective positions.

Again, a double-disk bandwidth chopper is placed right after the monolith at 6.5 m from the moderator. A double-disk frame overlap chopper is placed right after the bunker wall and the slit of the focusing position, at 16 m from source. An additional pair of choppers can be placed upstream of the objective lens to further suppress frame overlap. The chopper configuration can be seen in the time-distance diagrams of Fig. 6. The position sensitive detector is considered to have a surface area of 3.0 $\times$ 3.0 m^2^ and is placed at a fixed position matching the focal point of the objective lens at 29.5 m from the moderator. As in the case of ConvSANS, at this stage, there is no assumption of specific detector technology, which, however, requires sub-3 mm spatial resolution to fully exploit the focusing characteristics of the instrument.

### Instrument performance assessments

#### *Q*-ranges and *Q* resolutions

**Figure 7:**
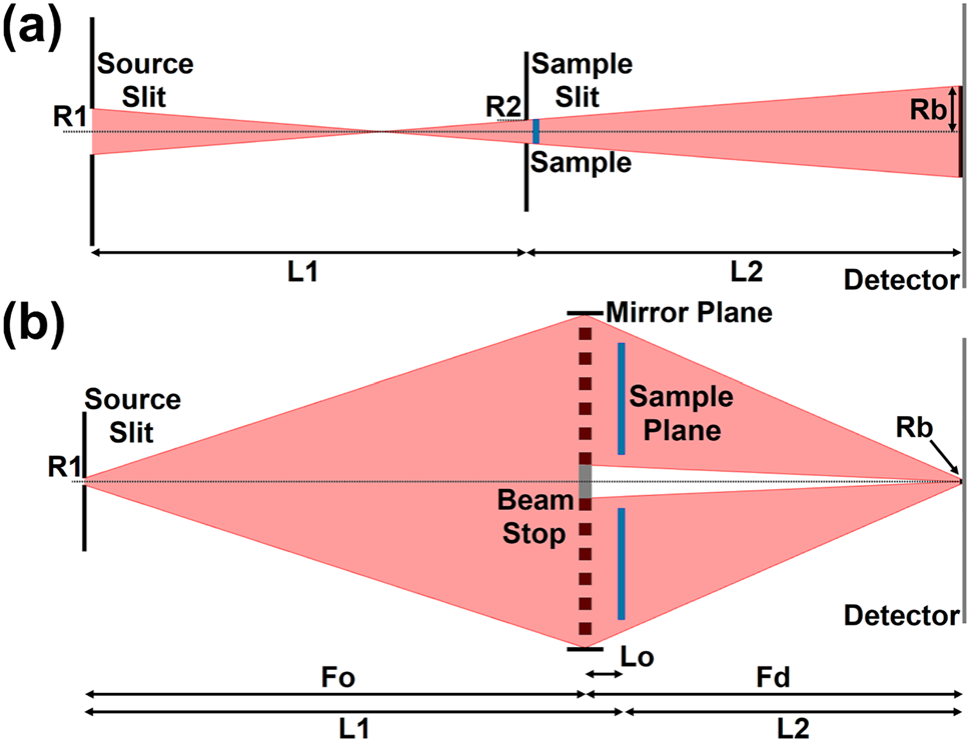
Schematic diagrams of the (**a**) conventional pinhole SANS geometry and (**b**) of the reflecting mirror SANS geometry. Note that the schematic dimensions are not comparable and are not on a common scale.

For initial evaluation of the instruments performance of each instrument concept, we calculate the $Q_{min}$ values as a function of sample size and the *Q* resolution variances, $\sigma ^2_Q$, based on the overall instruments geometries and wavelength bandwidths. Fig. 7 schematically depicts the classical pinhole geometry and a general objective lens geometry that are used for the calculations.

The $Q_{min}$ and $Q_{max}$ values are calculated using:

1a $$\begin{aligned} Q_{min}&= \frac{4\pi }{\lambda }\sin \left( \theta _{min}/2 \right) , \end{aligned}$$

1b $$\begin{aligned} Q_{max}&= \frac{4\pi }{\lambda }\sin \left( \theta _{max}/2 \right) , \end{aligned}$$

for a given wavelength, $\lambda$. $\theta _{min}$ and $\theta _{max}$ are the minimum and maximum detected scattering angles, respectively, given by:

2a $$\begin{aligned} \theta _{min} = \tan ^{-1}\left( \frac{Rb}{L2} \right) , \end{aligned}$$

2b $$\begin{aligned} \theta _{max} = \tan ^{-1}\left( \frac{DR}{L2} \right) , \end{aligned}$$

where *DR* is half the diagonal of the detector (assuming a rectangular geometry), and *L*2 is the sample-to-detector distance. *Rb* denotes the radius of the direct beam on the detector and is calculated by:

3a $$\begin{aligned} Rb = \left( \frac{L2}{L1} \right) \left( R1 + R2 \right) + R2, \end{aligned}$$

3b $$\begin{aligned} Rb = R1\left( \frac{Fd}{Fo} \right) = R1\left( \frac{L2 + Lo}{L1-Lo} \right) , \end{aligned}$$

for ConvSANS and WOF-SANS, respectively. *R*1 and *R*2 denote the radii of the source and sample apertures. For WOF-SANS the source aperture is considered to be the slit upstream of the objective lens (see Fig. 7). In both cases, *L*1 denotes the distance from the source aperture to the sample. In the case of WOF-SANS, *Lo* is the distance of the objective lens to the sample.

For a conventional pinhole geometry SANS instrument the *Q* resolution variance is given by^32^:

4 $$\begin{aligned} \sigma _{Q}^{2} = \left( \frac{2\pi }{\lambda L2} \right) ^2\left[ \left( \frac{L2}{L1} \right) ^2\frac{R1^2}{4} + \left( \frac{L1 + L2}{L1} \right) ^2\frac{R2^2}{4} + \frac{1}{3}\left( \frac{px}{2} \right) ^2 \right] + Q^2\frac{1}{12}\left( \frac{\Delta \lambda }{\lambda } \right) ^2, \end{aligned}$$

assuming a square (also called “box”) wavelength distribution, with *px* denoting the detector pixel size. Similarly, for a SANS instrument using focusing optics the *Q* resolution variance is given by^33^:

5 $$\begin{aligned} \sigma _{Q}^{2} = \left( \frac{2\pi }{\lambda L2} \right) ^2\left[ \left( \frac{L2+Lo}{L1-Lo} \right) ^2\frac{R1^2}{4} + \frac{1}{3}\left( \frac{px}{2} \right) ^2 \right] + Q^2\frac{1}{12}\left( \frac{\Delta \lambda }{\lambda } \right) ^2. \end{aligned}$$

**Figure 8:**
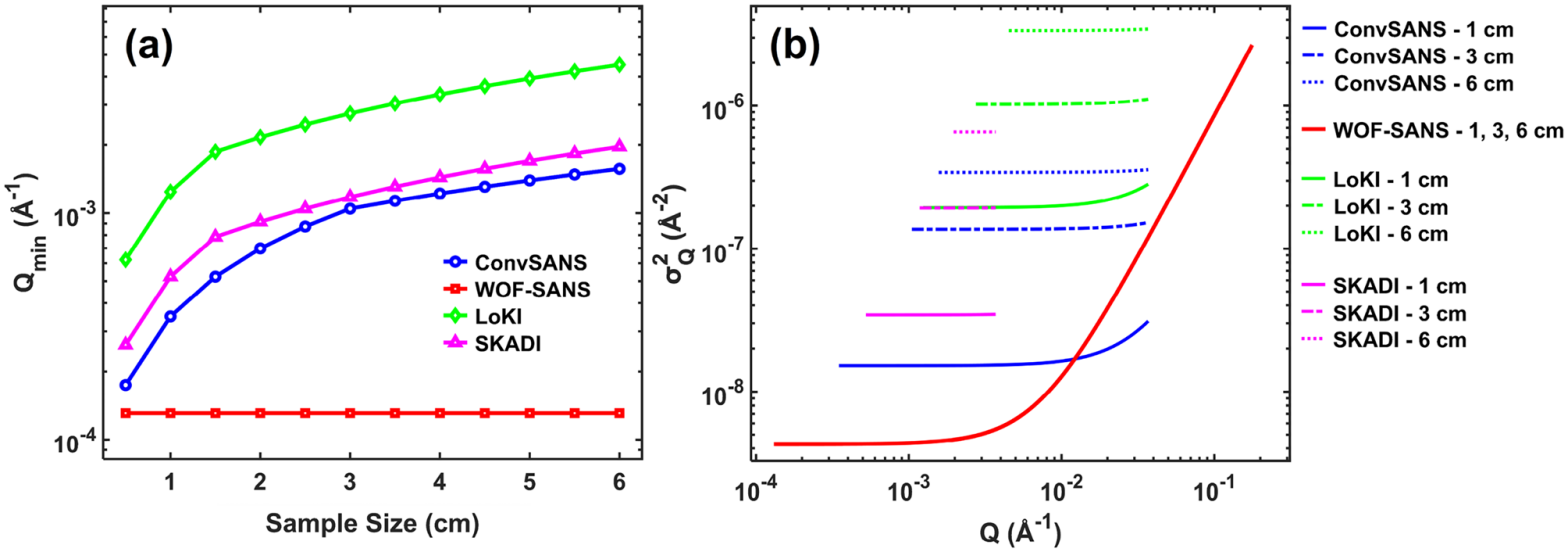
(**a**) Calculated $Q_{min}$ values for ConvSANS, WOF-SANS, LoKI, and SKADI as a function of sample size. (**b**) *Q* resolution variance, $\sigma ^2_Q$, as a function of scattering vector *Q* for ConvSANS and WOF-SANS in comparison to those of LoKI and SKADI. All resolutions were calculated for $\lambda _{max}$ = 12 Å and for the longest configuration of each instrument, on the rearmost detector (single detector for WOF-SANS).

Fig. 8a depicts $Q_{min}$ values as a function of sample size for ConvSANS, WOF-SANS, LoKi, and SKADI. Fig. 8b contains plots of $\sigma ^2_Q$ as a function of the scattering vector *Q* for the different instruments calculated on their rearmost detector and for three sample sizes of 1 cm, 3 cm, and 6 cm diameter. The LoKI and SKADI detector surface areas used for the calculations were 1.0 $\times$ 1.0 m^2^ and 0.2 $\times$ 0.2 m^2^, respectively (The rear detector of LoKI has dimensions of approximately 1.5 $\times$ 1.0 m^2^, however here we used 1.0  $\times$ 1.0 m^2^, which is the surface area to provide with the maximum possible symmetrical *Q* coverage). For WOF-SANS a fixed source aperture of $\text{\O }$ 4 mm was used with the sample assumed to be right at the objective lens (*Lo* = 0), positioned off-axis in order to be fully illuminated. A sample aperture with a size matching the sample size was assumed. To make direct comparisons of $Q_{min}$ values we considered the same maximum wavelength of 12 Å for all instruments. To simplify the calculations we assume no limitation by detector pixel sizes.

From our calculations, the WOF-SANS instrument exhibits the lowest $Q_{min}$ value among the instruments, which due to its focusing geometry (see also equations 1–3) is independent of sample size. Secondly, when we consider the three pinhole collimation instruments (ConvSANS, LoKI, and SKADI) the results align with expectations: the instrument featuring the tightest collimation yields the lowest $Q_{min}$. Regarding resolution, WOF-SANS excels across a significant portion of the *Q*-range, followed by ConvSANS, SKADI, and LoKI, in that respective order. WOF-SANS dominates at the low and middle *Q*-ranges while it appears to lose resolution at higher *Q* values, around and above 10^-2^ Å^-1^. This is due to the wavelength resolution contribution dominating at high *Q*; WOF-SANS, despite its superior geometrical resolution (apparent at low *Q*), as a very short instrument provides only moderate wavelength resolution. Furthermore, we see that the WOF-SANS instrument offers the largest dynamic *Q*-range, independent of sample size, an important attribute given its utilization of a single detector at a fixed position. This is a result of the instrument’s unique focusing geometry and a substantial wavelength bandwidth. It is important to note, however, that the overall $Q_{min}$ and resolution assessment for WOF-SANS assumes perfect mirrors and any deviation from this ideal scenario may introduce aberrations and other pitfalls that consequently might compromise both resolution and $Q_{min}$. The highest resolution in such focusing geometry is achieved when there is a substantial distance between the objective lens and the detector and the sample can be positioned close to the objective lens.

#### Monte Carlo ray tracing simulations and figure-of-merit

**Figure 9:**
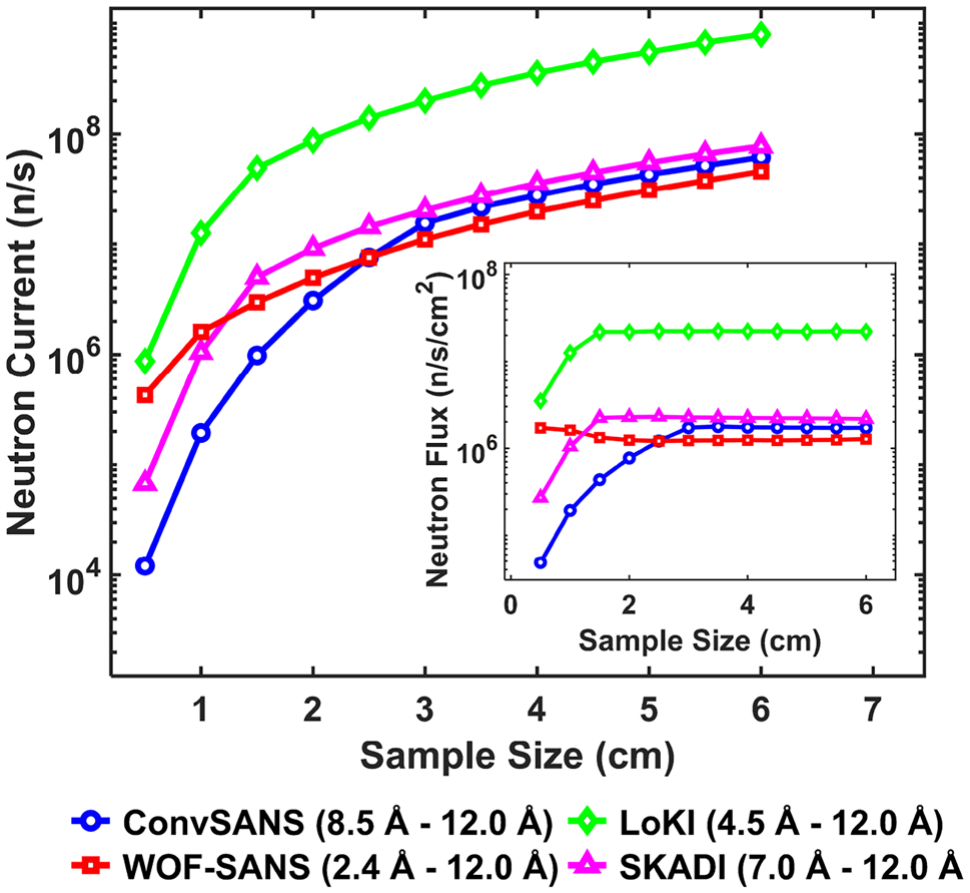
Total neutron current at the sample position as a function of sample size for ConvSANS, WOF-SANS, LoKI, and SKADI. Inset: total neutron flux at the sample position as a function of sample size.

To further evaluate the performance of the proposed instrument concepts we performed Monte Carlo ray tracing simulations using McStas^34,35^. As a moderator/neutron source we used a Monte Carlo Particle Lists (MCPL) input file^36^ that corresponds to the lower LD_2_ moderator design, with a viewable area of 15 $\times$ 15 cm^2^. To this end it was necessary to develop McStas modules for the used NMO, which could be successfully provided and are also contained in the 3.4 McStas release (https://www.mcstas.org/download/components/3.4_current/). Simulation results for WOF-SANS, ConvSANS, LoKI, and SKADI are given in Fig. 9, showing the total neutron intensity at the sample position as a function of sample size, with the total neutron flux given in the inset of the figure.

Determining the figure-of-merit (FOM) of a SANS instrument can be complex since there are many important parameters coming into play and of course different sets of samples and/or configurations need to be considered. For our SANS instruments, at this early stage of conceptual design, the useful parameters that can be considered are the total neutron intensity at the sample position (within a selected wavelength band), $Q_{min}$ and $Q_{max}$, as well as $\sigma ^2_Q(Q_{min})$. Thus, based on the FOM derived by information theory^13,37,38^, we define the following simple FOM formula:

6 $$\begin{aligned} FOM = \frac{I_{sample}}{\sigma ^2_Q(Q_{min})}\cdot ln\left( \frac{Q_{max}}{Q_{min}}\right) . \end{aligned}$$

**Figure 10:**
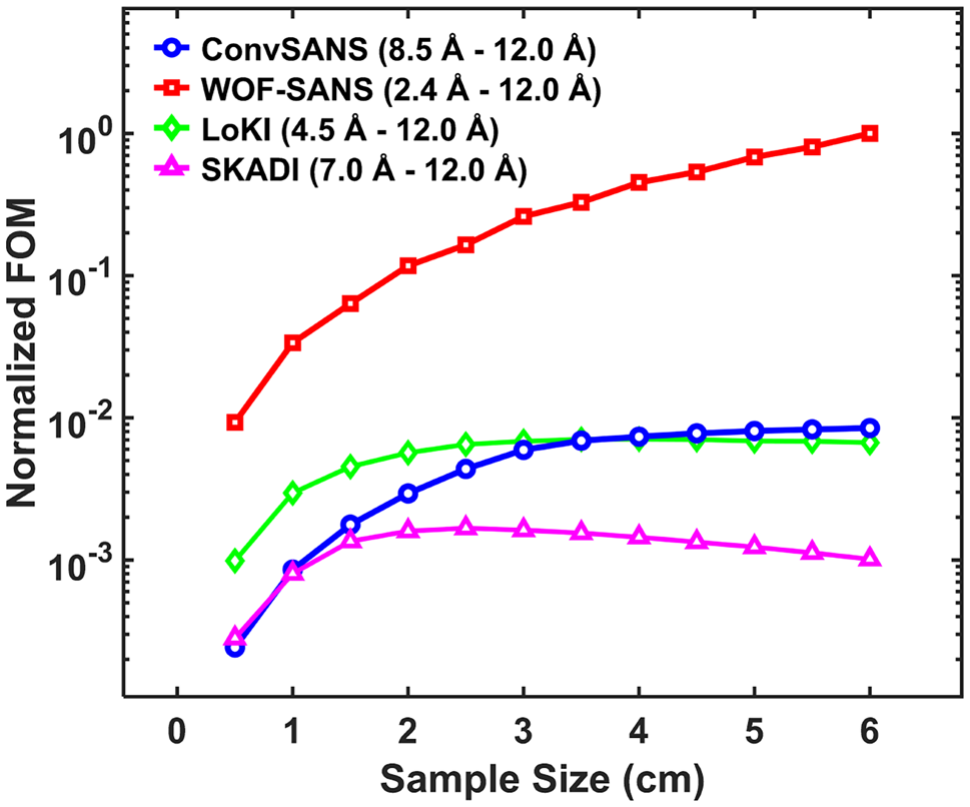
FOM of WOF-SANS, ConvSANS, LoKI, and SKADI as a function of sample size. The FOM is normalized by dividing each FOM curve by the maximum FOM value observed across all four curves, ensuring a relative comparison on a uniform scale.

With the above definition we see that the FOM is proportional to the intensity, as well as the ratio of $Q_{max}$ and $Q_{min}$, also highlighting the covered *Q* range. The *Q* resolution is inversely proportional to the FOM since higher resolution (i.e. lower resolution value) will increase the FOM. In Fig. 10 the FOM for both ConvSANS and WOF-SANS as a function of sample size is given, in comparison to the corresponding FOM calculated for LoKI and SKADI. Both $Q_{min}$ and $Q_{max}$ of each instrument were calculated on their rear detector (single detector for WOF-SANS) and considering the corresponding wavelength bands. Based on the calculations, the WOF-SANS instrument has a constant high performance compared to the other three instruments across all sample sizes, mainly attributed to its high $Q_{min}$ resolution and overall dynamic *Q* range as well as the large beam cross section at the considered sample position. The ConvSANS instrument, in spite of its relatively high resolution, exhibits a lower FOM than LoKI and WOF-SANS for smaller samples, however its performance improves and somewhat excels beyond LoKI when considering larger samples (>3 cm). ConvSANS and SKADI exhibit similar behavior at the smallest considered sample sizes, however, the FOM of SKADI decreases for larger samples (>2.5 cm), which can be mainly attributed to its overall small detector coverage and lower resolution, evident also in Fig. 8b.

## Neutron imaging

In the field of NI the proposed instrument concept complements the versatile approach of ODIN^1,15,16^ by providing a larger FoV with better homogeneity and notably a higher flux, in particular also in the cold neutron region. The higher flux enables better time resolution for operando studies as required today, e.g. in battery research, engineering and especially for additive manufacturing, which might also involve polarized neutron studies^39–42^. The cold neutron flux beyond the Bragg cut-off of important engineering materials, above all steels, enables enhanced sensitivity to density changes as required in many applications involving for example again manufacturing applications but also questions of hydrogen embrittlement etc. The large FoV and superior homogeneity support accurate measurements of large specimens as required in many industrial applications but also for cultural heritage objects and large devices^43–46^. The instrument is short compared to ODIN, which focuses on time-of-flight imaging applications utilizing different wavelength resolutions. Thus, the instrument proposed here offers very relaxed wavelength resolution, but a broad bandwidth, which can be accessed by adding bandwidth choppers to the setup. While such installations have not been considered in these preliminary simulation models, back-of-the-envelop calculations show that the instrument would support a number of applications in this regime from crystalline phase mapping to dark-field contrast imaging, probing apart from macroscopic structures also microscopic structures through small-angle scattering analysis^47–51^. Furthermore, when considering to realize a long instrument cave, also the use of NMO, such as Wolter optics should be considered to leverage corresponding flux gains at high resolution for small samples as would be enabled by a microscopy-type setup with a condenser and objective lens^12^.

### Conceptual design

**Figure 11:**
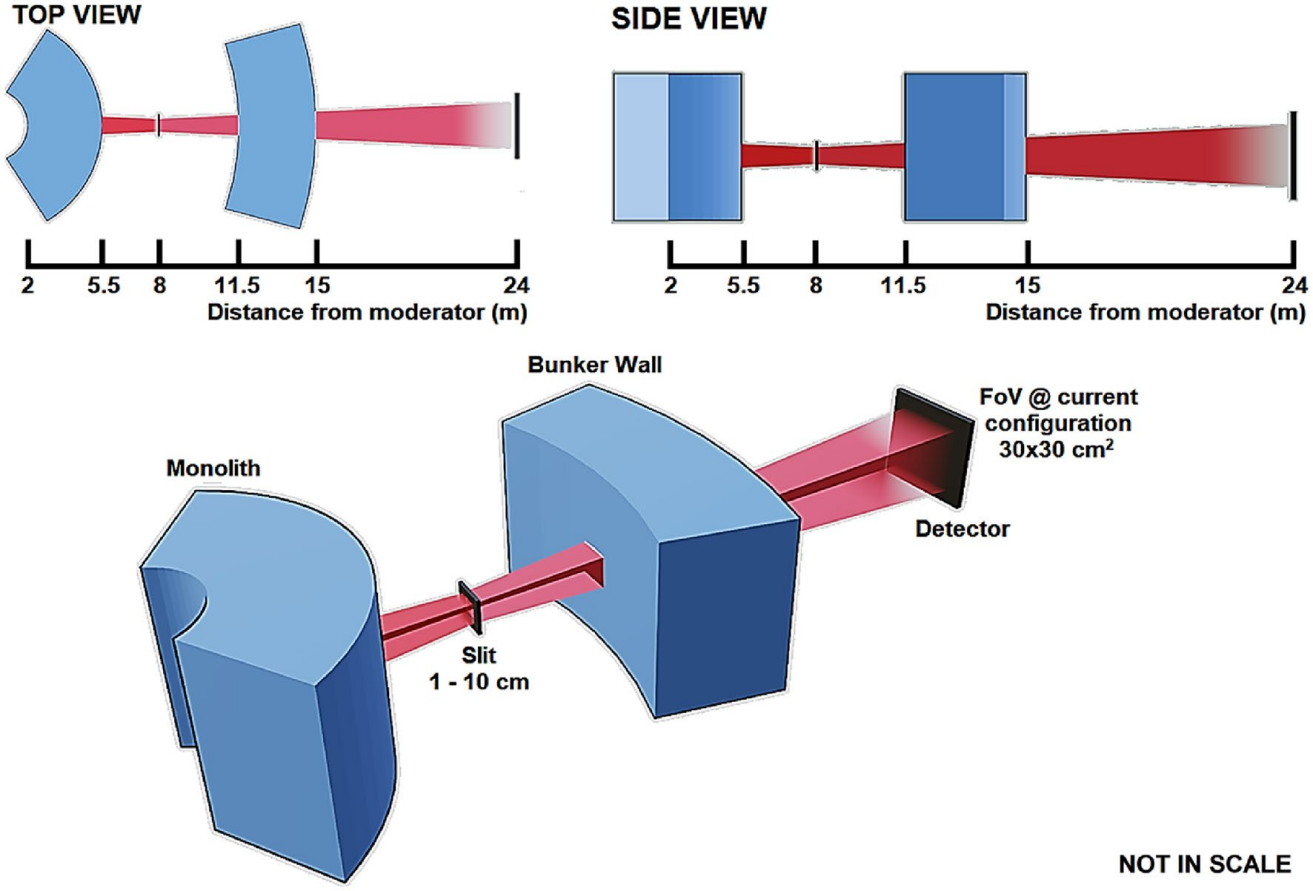
Schematic overview of the NI instrument design in isoprojection, as well as from top and side view.

The NI instrument design presented here utilizes a conventional pinhole geometry. The instrument takes advantage of the large-surface moderator to have increased flux at the sample position with large FoV, without the use of any neutron guides, which offers good beam homogeneity. The neutron beam is travelling through beam tubes with absorbing coatings through the ESS monolith and bunker wall. An adjustable aperture is placed 8 m from the moderator. A nominal detector is placed 16 m from the aperture (24 m from the moderator) having a surface area of 0.4 $\times$ 0.4 m^2^, however, the choice of dimensions is arbitrary and different detectors could be employed depending on the needs of each experiment. A longer cave and the setup of a neutron microscope with NMO would potentially require a more downstream detector position with a smaller FoV. A schematic of the conceptual neutron imaging instrument design can be seen in Fig. 11.

In the current setup, we can define the ratio *L*/*D* with *L* being the distance between the aperture and the sample and *D* the aperture size/diameter. This ratio defines the geometric resolution capability of the configuration and is also used to calculate the geometric blur of the sample, *d* = *l*/(*L*/*D*), in the image (detector) plane, where *l* is the distance between the sample and the detector. For example, with *L* = 16 m, *D* = 5 cm, and *l* = 1 cm, *L*/*D* = 320 and the image blur *d* = 0.0031 cm, i.e. 30 $\upmu$m.

### Instrument performance assessments

#### Monte Carlo ray tracing simulations and figure-of-merit

**Figure 12:**
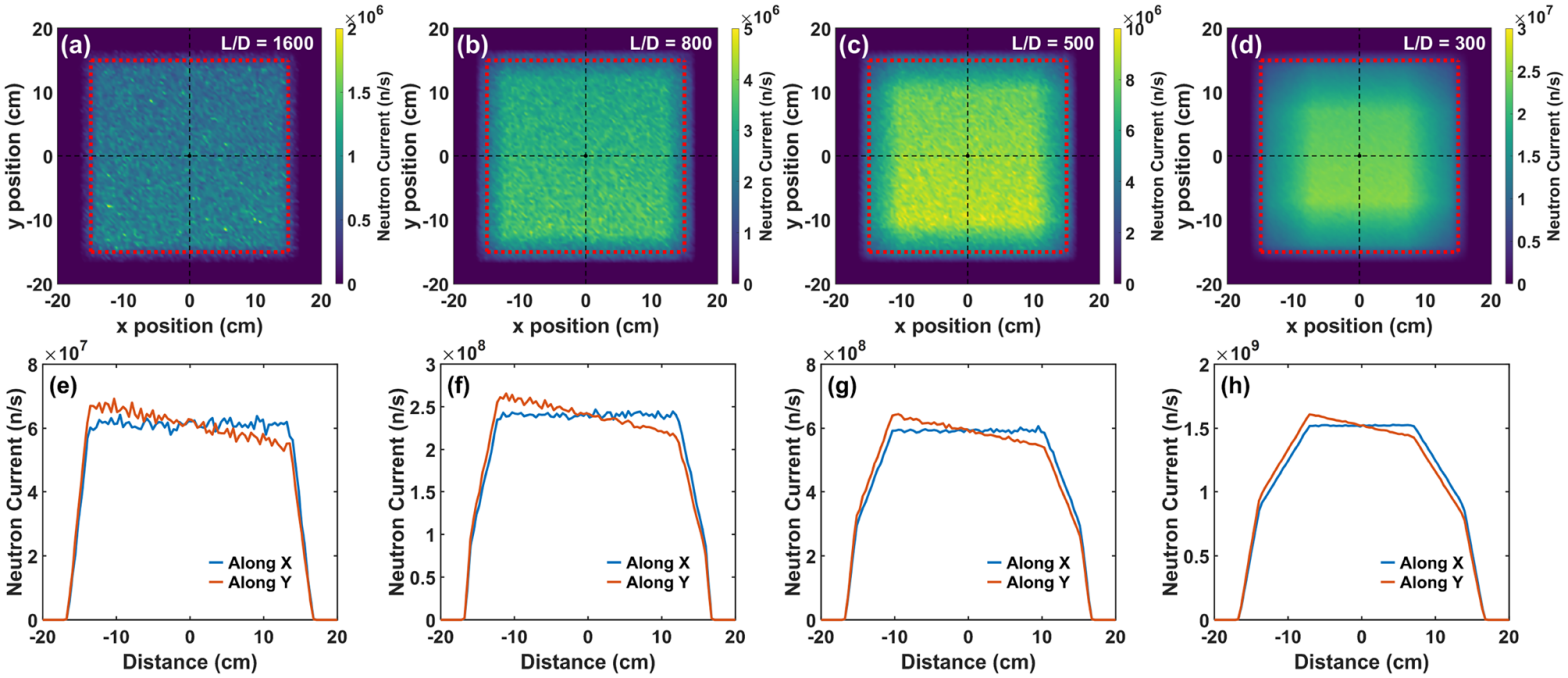
(**a**–**d**) Simulated detector images of the NI instrument for *L*/*D* = 1600, 800, 500, and 300. The red dotted box indicates the nominal FoV. (**e**–**h**) Line profiles across the detector images along both the vertical and horizontal directions.

Similarly to the SANS instruments, Monte Carlo Ray Tracing simulations using McStas were performed to investigate the performance of the NI instrument, mainly in terms of FoV, beam homogeneity and flux. For the simulations, we used the same moderator input component as for the SANS simulations. The geometry of the instrument was defined as described above, with the detector fixed at 24 m from the moderator and 16 m from the slit position, thus expecting a nominal FoV of about 30 $\times$ 30 cm^2^, i.e. twice the surface area of the moderator. The slit size was varied to test the performance of the instrument for different *L*/*D* values. Example detector images for select *L*/*D* values along with corresponding line profiles across the detector images can be seen in Fig. 12.

**Figure 13:**
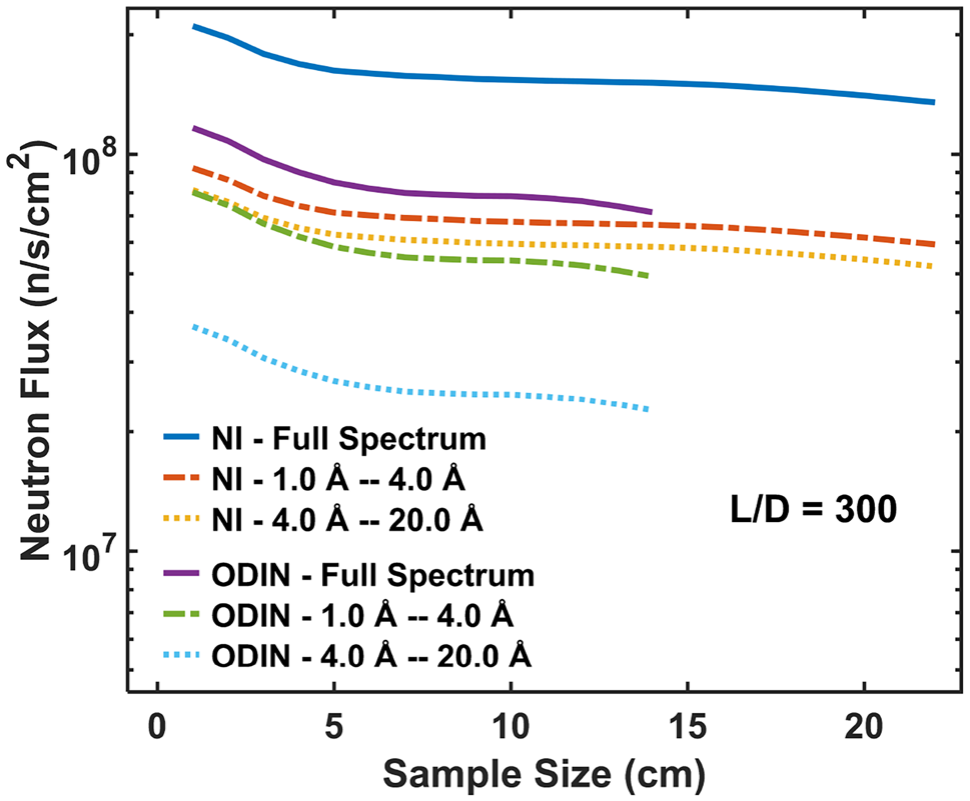
Total neutron flux at the sample position for both NI and ODIN as a function of sample size for *L*/*D* = 300 and for three spectral regions: full spectrum, 1– 4 Å, 4–20 Å. For the flux calculations we assumed an effective FoV matching the sample size.

The red frame in each detector image indicates the nominal FoV. From the images, as expected, we see that the intensity is increasing with increasing slit size, i.e. decreasing *L*/*D* ratio. The FoV remains relatively homogeneous for large *L*/*D* however at *L*/*D* = 500 and below a decrease of intensity in the outer FoV regions is observed which reduces the effective FoV due to the penumbra created by the geometry. Fig. 13 shows the simulated neutron flux of the NI instrument at the sample/detector position, as a function of sample size, for *L*/*D* = 300 and for three spectral regions. The total neutron flux of ODIN for the same *L*/*D* and the same spectral regions is also plotted for comparison. The maximum sample size for each instrument was chosen where the flux is at about 75 % peak flux in the penumbra, while only the FoV defined by the sample size is used for the flux calculations. The observed higher flux of the NI instrument at the second moderator is attributed to its simple and short geometry and for long wavelengths also to the corresponding characteristics of the moderators.

For a better characterization of performance of the NI instruments we again define a FOM. For an imaging instrument the main factors contributing to the FOM are the following. The available intensity that contributes linearly to the FOM as it defines the required exposure time with regards to achieving a good signal-to-noise ratio. The intensity that is taken as the intensity *I* in the region of the FoV with the average incident flux. The FoV also contributes linearly to the FOM through enabling measurements of objects of corresponding size. The capability for spatial resolution is given by the collimation ratio *L*/*D*. As the *L*/*D* ratio impacts the flux by (*D*/*L*)^2^, the collimation adds a factor of (*L*/*D*)^2^ to the FOM. An inhomogeneous flux distribution is adversely affecting the imaging quality, in particular in the case of significant minima in the beam cross section, which are typical for the provision of a beam through a neutron guide due to gaps in the transported divergence. A simple factor taking all aspects of such artifacts into account is difficult to define and has never ultimately been defined. Here, for simplicity, we consider the standard deviation over the FoV to contribute to the FOM as the inverse of its square root. The full expression for the FOM of an imaging instrument used in this work can thus be written as:

7 $$\begin{aligned} FOM = I_{mean}\cdot FoV \cdot \left( {\frac{L}{D}}\right) ^2\cdot \sqrt{\frac{1}{std(I)}}. \end{aligned}$$

**Figure 14:**
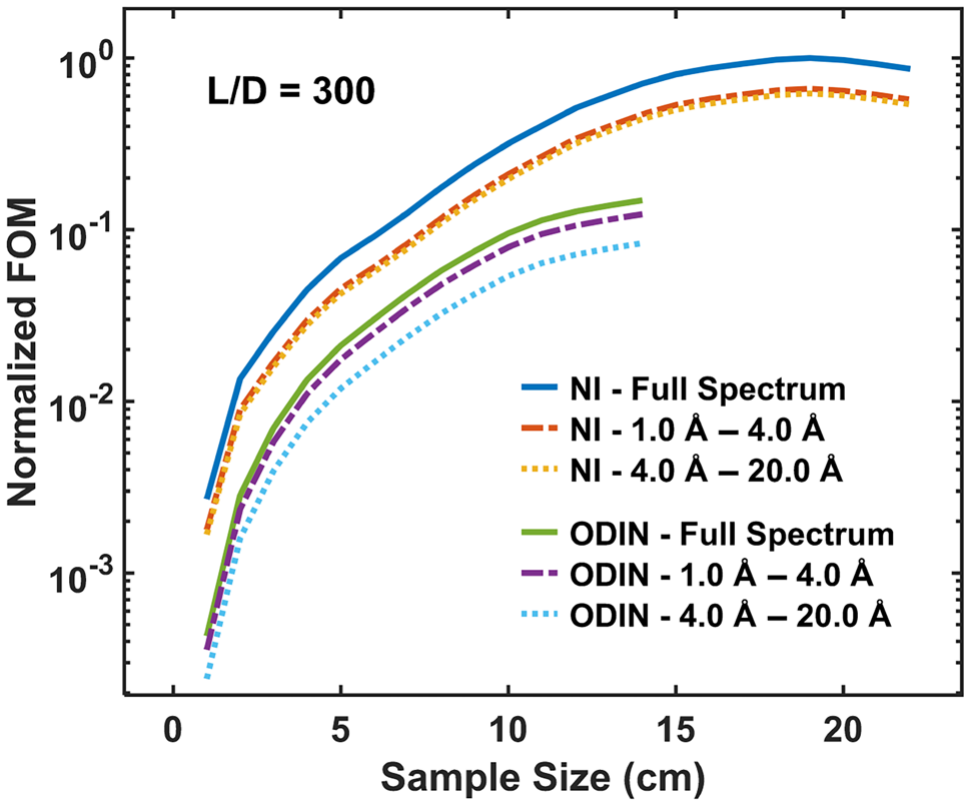
FOM of NI and ODIN as a function of sample size. The calculations were done for *L*/*D* = 300 and for three spectral regions: full spectrum, 1–4 Å, 4–20 Å. Only the FoV defined by the sample size is used for the FOM calculations. The FOM is normalized by dividing each FOM curve by the maximum FOM value observed across all six curves, ensuring a relative comparison on a uniform scale.

The resulting FOM for the conceptual NI instrument at the second moderator as a function of sample size is given in Fig. 14. The calculated FOM of ODIN is also plotted for comparison. For both instruments the calculations were done for the three spectral regions given in the figure and for *L*/*D* = 300. Only the FoV defined by the sample size is used as basis for the calculations and as in Fig. 13 the maximum sample size for each instrument was chosen where the flux is at about 75 % peak flux in the penumbra. Evidently, in both cases we see that the FOM increases, according to its definition with respect to the FoV, with increasing sample size up to a point where it stagnates and then drops distinctly, which is where it is affected by the penumbra region. For ODIN this occurs at about 14 cm sample size, matching its nominal maximum FoV (as in Fig. 13). For the NI instrument considered for the second moderator a decrease in the FOM is seen significantly later at about 20 cm sample size, which resonates with the observed penumbra in Fig. 12d.

Note that the presented conceptual design to date only includes the basic beamline, which, however, has potential for a multitude of imaging techniques and optional equipment to be installed. Apart from the mentioned potential for a NMO-based neutron microscope and wavelength band choppers, also gratings and polarized neutron components have to be considered when moving towards a full instrument design taking advantage of our base layout.

## Conclusion

We have presented a study of conceptual designs for neutron instruments for condensed matter research tailored to capitalize on the potential of a second, large-surface moderator at the European Spallation Source. The envisioned instruments, with a focus on two alternative SANS instrument concepts and a NI instrument concept, aim to leverage the anticipated high intensity and large surface which the second moderator is designed to offer. The instruments show the potential to excel over and complement the currently constructed instruments in different use cases.

Simulations and corresponding FOM considerations demonstrate that the SANS instrument concept based on a conventional pinhole geometry has an advantage considering large samples while the SANS instrument based on NMO excels in resolution and $Q_{min}$ values, leading to a favorable FOM for a wide range of applications. The NI instrument concept excels in providing a large homogeneous FoV, with moderate flux gains for conventional and low wavelength resolution techniques. The envisioned instruments, taking advantage of the second, large-surface moderator, hold potential to enhance the capabilities and versatility of neutron scattering at the European Spallation Source. It has to be noted that these conceptual considerations also underline a great potential held by NMO and the further development of such, beyond the presented simulation tools, needs to be pursued in order to harness their expected impact on neutron instrumentation.

## Data Availability

The datasets used and/or analysed during the current study available from the corresponding author on reasonable request.
